# Numerical Simulation of Fatigue Damage in Cross-Ply CFRP Laminates: Exploring Frequency Dependence and Internal Heat Generation Effects

**DOI:** 10.3390/polym17030432

**Published:** 2025-02-06

**Authors:** Natsuko Kudo, M. J. Mohammad Fikry, Shinji Ogihara, Jun Koyanagi

**Affiliations:** 1 Department of Materials Science and Technology, Graduate School of Tokyo University of Science, 6-3-1 Niijuku, Katsushika-ku, Tokyo 125-8585, Japan; 8219b01@alumni.tus.ac.jp; 2 Department of Mechanical Engineering, The University of Akron, 244 Sumner St., Akron, OH 44325-3903, USA; mfikry@uakron.edu; 3 Department of Mechanical and Aerospace Engineering, Tokyo University of Science, 2641 Yamazaki, Noda-shi, Chiba-ken 278-8510, Japan; ogihara@rs.tus.ac.jp; 4 Department of Materials Science and Technology, Tokyo University of Science, 6-3-1 Niijuku, Katsushika-ku, Tokyo 125-8585, Japan

**Keywords:** CFRP cross-ply laminate, frequency dependence, fatigue damage, numerical simulation, internal heat generation

## Abstract

A numerical simulation investigating the frequency dependence of fatigue damage progression in carbon fiber-reinforced plastics (CFRPs) is conducted in this study. The initiation and propagation of transverse cracks under varying fatigue test frequencies are successfully simulated, consistent with experiments, using an enhanced degradable Hashin failure model that was originally developed by the authors in 2022. The results obtained from the numerical simulation in the present study, which employs adjusted numerical values for the purpose of damage acceleration, indicate that the number of cycles required for the formation of three transverse cracks was 174 cycles at 0.1 Hz, 209 cycles at 1 Hz, and 165 cycles at 10 Hz. Based on these results, it is demonstrated that under high-frequency cyclic loading, internal heat generation caused by dissipated energy from mechanical deformation, attributed to the viscoelastic and/or plastic behavior of the material, exceeds thermal dissipation to the environment, leading to an increase in specimen temperature. Consequently, damage progression accelerates under high-frequency fatigue. In contrast, under low-frequency fatigue, viscoelastic dissipation becomes more pronounced, reducing the number of cycles required to reach a similar damage state. The rate of damage accumulation initially increases with test frequency but subsequently decreases. This observation underscores the importance of incorporating these findings into discussions on the fatigue damage of real structural components.

## 1. Introduction

Carbon fiber-reinforced plastics (CFRPs) are widely used in aerospace, automotive, and structural applications due to their high strength-to-weight ratio, durability, and excellent mechanical properties [1]. Among their critical performance aspects, fatigue behavior plays a key role in ensuring long-term reliability [2], making it essential to understand fatigue damage for accurate performance prediction and longevity in practical applications. Numerous studies have advanced this field through experimental investigations and empirical modeling efforts. Aoki et al. [3,4] demonstrated the importance of intra-laminar and inter-laminar damage models by simulating progressive damage in CFRP laminates and assessing the residual strength of thin-ply laminates under tensile fatigue loading. Brod et al. [5] and Brunbauer and Pinter [6] examined the effects of stress states and fiber volume content on fatigue-induced damage mechanisms, highlighting the interplay between material composition and loading conditions. Hosoi et al. [7] quantitatively evaluated fatigue damage progression, reinforcing the idea that applied stress levels significantly influence damage growth. Panella and Pirinu [8] extended these findings by analyzing fatigue and damage behavior in aeronautical CFRP elements under tension and bending loads, emphasizing the need for tailored analytical approaches.

Advanced monitoring techniques, such as acoustic emission, have also been employed to enhance the understanding of fatigue mechanisms. Guo et al. [9] used acoustic emission monitoring to identify damage modes under fully reversed loading, whereas Just et al. [10] explored the influence of reversed fatigue loading on damage evolution, revealing critical insights into CFRP fatigue behavior. Longbiao [11,12] introduced hysteresis dissipated energy-based parameters to monitor damage and predict fatigue life in CFRP composites, providing a novel approach for studying fatigue progression. Skinner et al. [13] investigated biaxial loading conditions in carbon fiber composites, and Xu et al. [14] demonstrated self-sensing capabilities for monitoring fatigue damage in bridge deck components made with cementitious carbon fiber composites. Ogi et al. [15,16] developed empirical models for matrix cracking in cross-ply laminates under static and cyclic fatigue loadings, elucidating the mechanism of transverse cracking phenomena. Yashiro and Okabe [17] showcased the application of embedded fiber Bragg grating sensors for estimating fatigue damage in holed composite laminates. Kitagawa et al. [18,19] further expanded on these studies, focusing on transverse crack multiplication and evaluating the fatigue properties of carbon fiber/epoxy matrix interfaces.

Transverse cracking in CFRP laminates remains a key area of research, with Deng et al. [20] offering one of the most recent advancements in this field. Using the entropy-based Hashin law [21], they modeled the natural progression of transverse cracks by linking strength degradation to entropy, defined as the dissipated energy divided by temperature. This model incorporated a Weibull distribution to account for variability in the strength of individual elements. Their numerical simulations closely matched experimental observations, achieving high-fidelity modeling. However, the dependence of the results on frequency during cyclic loading has not been addressed. Basically, under high-frequency cyclic loading, internal heat generation caused by dissipated energy from mechanical deformation—attributed to the viscoelastic and/or plastic behavior of the material—exceeds thermal dissipation to the environment, leading to an increase in specimen temperature. Consequently, damage progression accelerates under high-frequency fatigue. In contrast, under low-frequency fatigue, viscoelastic dissipation becomes more pronounced, reducing the number of cycles required to reach a similar damage state. Overall, the rate of damage accumulation initially increases with test frequency but subsequently decreases. This issue is highly complex, and several reports, primarily experimental studies, have addressed it [22,23,24,25,26,27,28,29,30,31]. Deng et al. [20] demonstrated that lower frequencies reduce the number of cycles required for transverse cracks to appear, but this represents the extent of their explanation. A critical concern is that frequencies are generally selected for durability evaluation tests based on practical reasons—higher frequencies to shorten the testing time or slightly lower frequencies to avoid complications caused by temperature rise. As a result, there is a risk that durability evaluation tests are conducted under conditions that yield the highest apparent durability, potentially leading to overly optimistic results.

Considering this background, the frequency dependence of fatigue failure has recently been investigated, assuming a single material [32]. A fatigue failure model was proposed for a resin material based on the entropy damage law [33,34,35,36,37,38], which has been increasingly applied in fatigue studies in recent years. In our previous works, fatigue failure was simulated using the entropy damage code developed for resin in conjunction with the finite element software ABAQUS, while accounting for heat generation and dissipation. The model calculates dissipated energy from mechanical loading, normalizes it by temperature to derive entropy, and correlates it with the progression of resin damage, as demonstrated in our micro-scale numerical simulations of fatigue failure in CFRP [39]. This foundational study is complemented by our application of a viscoelastic entropy damage criterion to short-fiber-reinforced plastics, highlighting the critical role of viscoelastic behavior in fatigue response [40]. Further advancements were made by treating dissipated energy as a heat source, integrating it into the simulation of heat generation, an essential aspect emphasized in our durability analysis of CFRP adhesive joints using entropy damage modeling [41]. The model’s predictive capabilities were validated through our work on residual strength prediction in unidirectional CFRP, where a nonlinear viscoelastic constitutive equation incorporating entropy damage was employed [42]. Specifically, at low frequencies, the number of cycles to failure decreases, while at higher frequencies, it initially increases but ultimately declines due to significant heat generation effects. This observation was explored in our efficient fatigue damage prediction in carbon fiber composites using multi-timescale analysis and machine learning [43]. Finally, our investigation into the durability of viscoelastic polymer materials under variable loading conditions further underscores the importance of considering variable loading effects in fatigue analysis [44]. Although our previous work focused on a single resin material and CFRP with a smaller scale and simpler configurations, it represents a state-of-the-art approach to modeling frequency dependence in fatigue.

Building on this, the modeling of frequency dependence in CFRP remains unexplored, and the present study aims to address this gap. First, we briefly explain the entropy-based Hashin law [21], which can be used to represent the fatigue failure of CFRP. Strength degradation is governed by dissipated energy divided by temperature, though the specific mechanism of how strength decreases remains unclarified. Sekino et al. [45] have partially explored this aspect. Fundamentally, the model accurately translates microscale phenomena to the mesoscale, providing a reasonable representation of strength degradation as a function of loading history. In the entropy-based Hashin law, dissipated energy is calculated during the process, and this dissipated energy is considered the origin of the temperature rise observed in actual materials, a concept also utilized in [32]. By assigning two roles to dissipated energy—strength degradation and temperature rise—the objectives of the present study can be achieved. Temperature rise is caused by viscoelastic dissipation, generating heat that flows through the specimen and dissipates from the surface, resulting in temperature distribution. In regions with higher temperatures, time accelerates according to the time–temperature superposition principle, further increasing heat generation. Capturing these coupled effects should enable more comprehensive simulations of the phenomenon.

In this study, we expanded the constitutive model proposed by Kudo et al. [32] to composite materials and incorporated the entropy-based Hashin law [21] to analyze fatigue damage progression in CFRP laminates. By plotting the number of cycles that occur before the appearance of several transverse cracks on the vertical axis and the test frequency on the horizontal axis, we observed the expected behavior: an initial increase in the number of cycles with frequency, followed by a decrease. Some studies clearly define the ranges for low and high frequencies in fatigue tests [46,47]. However, since this study is a qualitative evaluation, only relatively low and high frequencies were considered. This study involved a qualitative comparison between the model and experimental results, demonstrating the practical assessment of fatigue damage behavior in CFRP laminates. Notably, it is possible to simulate scenarios where transverse cracks originate in the warmer interior rather than the cooler edges, highlighting a significant difference between static and fatigue testing in terms of crack initiation patterns. Since this study qualitatively examines how damage progression changes with different frequencies, achieving the observed trend within a time-accelerated simulation space fulfills the research objective. A more detailed quantitative comparison with experimental data will be addressed in future studies.

## 2. Numerical Procedure

### 2.1. Viscoelastic Constitutive Law

**Figure 1:**
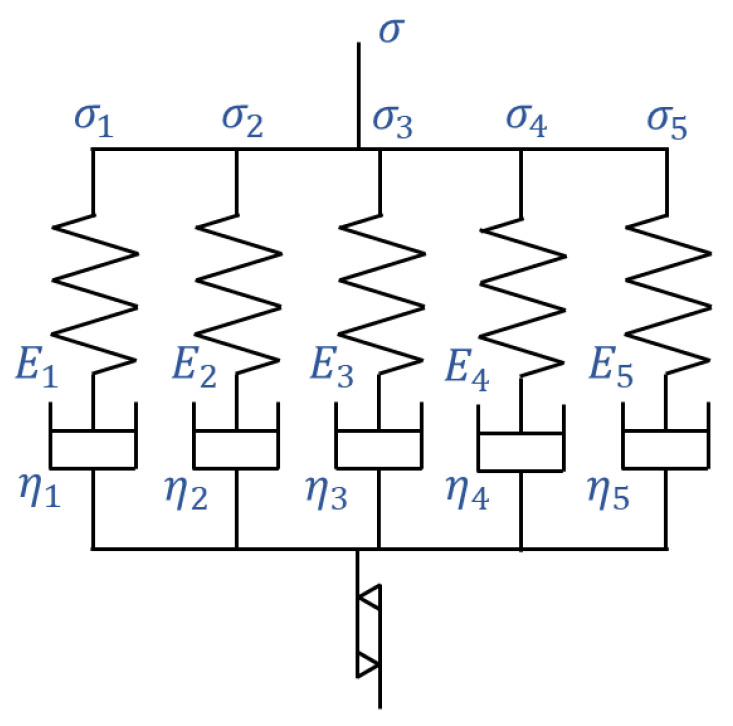
A generalized Maxwell model consisting of five parallel Maxwell elements and a non-recoverable strain element.

The stress in the material is calculated using a viscoelastic model to accurately represent the time-dependent mechanical behavior. A constitutive equation is initially derived to describe the material behavior only up to the point of damage initiation. Figure 1 illustrates the generalized Maxwell model used to analyze viscoelastic behavior in this study. This configuration relies on five independent elements to collectively capture a wide range of material behaviors. The stress tensor is formulated as follows:

(1) $$\sigma_{ij}\left(t\right)=\int_{0}^{t}E_{ijkl}^{r}\left(t-t^{\prime }\right)\frac{d\varepsilon_{kl}^{ve}}{dt^{\prime }}dt^{\prime }$$

where $\varepsilon_{kl}^{ve}$ represents the viscoelastic strain, and $E^{r}$ is the relaxation modulus. The relaxation modulus, $E^{r}$, is expressed as a summation of five Maxwell elements (Figure 1):

(2) $$E^{r}(t)=\sum_{i=1}^{5}E_{ijkl}\exp\left(-\frac{tE_{i}}{\eta_{i}}\right)$$

where $E_{i}$ is the elastic modulus, and $\eta_{i}$ is the viscosity coefficient of the i-th element. There are finite deformation and nonlinear viscoelastic constitutive theories that incorporate a nonlinear dashpot and have been conventionally developed [48,49]. However, in this study, we use five independent linear dashpots to better capture the complex viscoelastic response, especially under changing temperature conditions. Using multiple elements provides a more detailed representation of the material’s behavior by accounting for its history of cyclic loading and its orthotropic nature, including key considerations such as the Hashin damage criterion, strength degradation, and thermal effects, which will be explained in later sections [21,32].

A non-recoverable strain element, as proposed in [32], is essential for calculating temperature elevation by incorporating the material’s frequency-dependent characteristics. Therefore, a non-recoverable strain element is introduced in this study (Equation (3)), as illustrated in Figure 1.

(3) $$\Delta \varepsilon^{nr}\left(i\right)=\left(a\times {\sigma (i)}^{c}\exp\left(-{\left(\frac{\sigma (i)}{\sigma_{0}}\right)}^{d}\right)+b\left(1-\exp\left(-{\left(\frac{\sigma (i)}{\sigma_{0}}\right)}^{d}\right)\right)\right)\times \Delta \varepsilon \left(i\right)$$

Here, $\Delta \varepsilon^{nr}$ and Δε denote the non-recoverable and total strain increments, respectively, while a, b, c, and d are fitting constants. The non-recoverable strain increment is influenced by the stress in each direction, with the first term being proportional to the c-th power of the stress and the second term scaled by the constant b. These terms are linked through the constant $\sigma_{0}$, which incorporates the stress into the overall formulation. This equation is based on the formulation used in [32]. Herein, the rule of mixtures from [21] is applied to separate the stress in the composite material from the stress in the resin. The non-recoverable strain of the resin is then calculated, and the rule of mixtures is used again to determine the non-recoverable strain of the composite material (elements). The material constants used for this procedure were given in [21].

**Figure 2:**
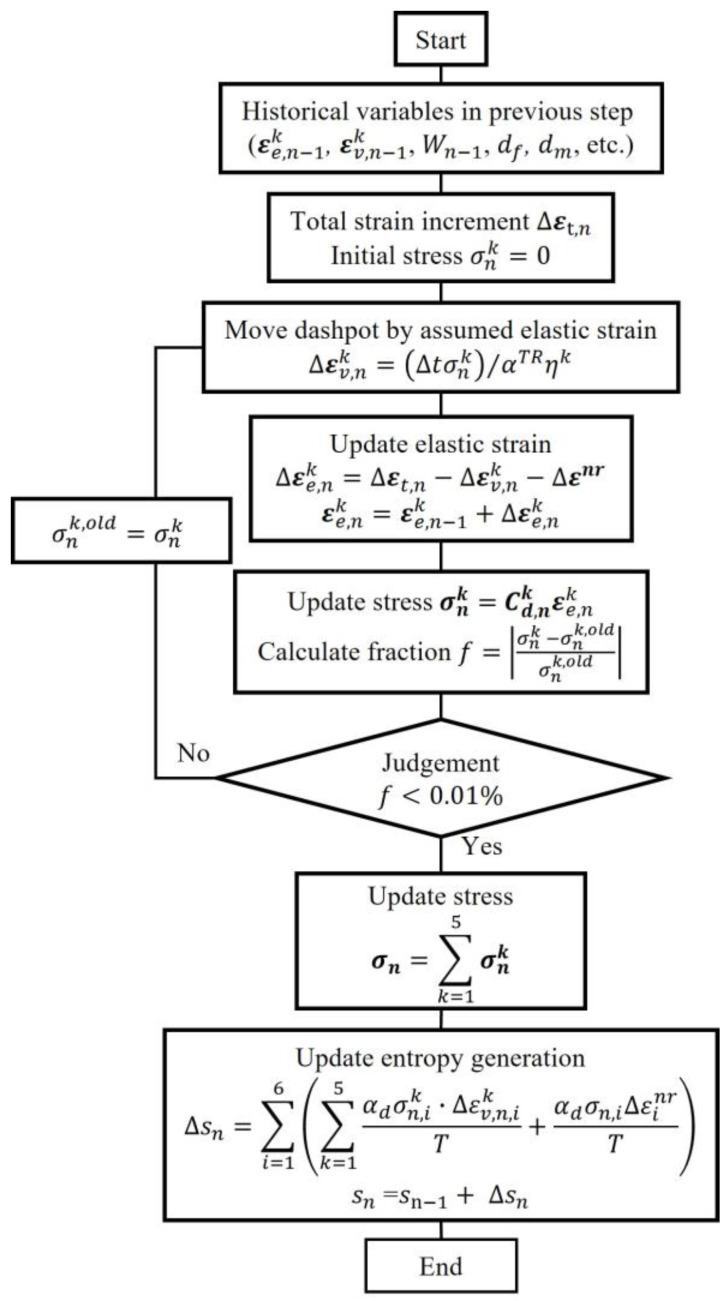
Flowchart of process used for updating stress and entropy.

The viscoelastic model, based on an entropy-based failure criterion, is implemented in the finite element analysis software Abaqus (Abaqus 2021 Standard) through a user-defined UMAT subroutine. Figure 2 shows a flowchart detailing the process for updating stress and entropy. As shown in the entropy generation equation in the figure, a time acceleration factor $\alpha_{d}$ is introduced to expedite the simulation process, primarily because of computational cost constraints, which made it impractical to simulate full-scale loading conditions. The factor has no other physical significance other than the above purpose.

### 2.2. Heat Conduction and Heat Release

To account for temperature variations in the simulation, heat transfer from the surroundings to the material is described by the following equation:

(4) $$Q=Ah\left(T_{el}-T_{atm}\right)$$

where Q represents the heat flux, A is the surface area of the boundary where heat transfer occurs, and h is the heat transfer coefficient, which is set to 10 W/(m^2^·K) in this study [50]. $T_{el}$ and $T_{atm}$ are the element temperature and surrounding temperature, respectively. Heat conduction within the material is modeled using the following equation:

(5) $$c\rho \frac{\partial T}{\partial t}=\lambda_{x}\frac{\partial^{2}T}{\partial x}+\lambda_{y}\frac{\partial^{2}T}{\partial y}+\lambda_{z}\frac{\partial^{2}T}{\partial z}+\alpha_{d}\frac{\partial E_{dis}}{\partial t}$$

where c denotes the specific heat, ρ represents the density, λ corresponds to the thermal conductivity, T is the temperature, and $E_{dis}$ is the dissipated energy resulting from the viscoelastic and non-recovery deformations:

(6) $$E_{dis}=\sigma_{n}:∆\varepsilon_{v}+\sigma_{n}:∆\varepsilon^{nr}$$

Here, $\sigma_{n}$ represents the stresses, $∆\varepsilon_{v}$ represents the viscoelastic strain increments, and $∆\varepsilon^{nr}$ represents the non-recovery strain increments.

In this study, the time–temperature superposition principle [32] is applied. The Arrhenius equation is adopted to model the material’s behavior:

(7) $$\log\alpha^{TR}\left(T^{test}\right)=\frac{\Delta H}{2.303R}\left(\frac{1}{T^{test}}-\frac{1}{T^{ref}}\right)$$

where $\alpha^{TR}$ is the shift factor, ΔH is the activation energy, R is the gas constant (8.314 $\times \;10^{-3}$ kJ/(K·mol)), $T^{ref}$ is the reference temperature, and $T^{test}$ is the testing temperature. The material properties of the Maxwell elements are characterized at the reference temperature. Given that the simulation involves a temperature rise, the resin response at the testing temperature is calculated while incorporating the shift factor to correlate the properties between the test and reference temperatures. The values of activation energy and reference temperature adopted in this study are 150 kJ/mol and 300 K, respectively. The relationship governing the times at the test and reference temperatures can be written as

(8) $$t_{T^{test}}=t_{T^{ref}}\times \alpha^{TR}\left(T^{test}\right)$$

The time-dependent flow in the Maxwell model is represented by the stress in the dashpot, calculated using the viscosity coefficient η and the strain increment $d\varepsilon_{v}$:

(9) $$\sigma =\eta \frac{d\varepsilon_{v}}{dt}$$

Based on Equation (8), the relationship between time increments at the reference and test temperatures is given by

(10) $$dt\left(T^{ref}\right)=\frac{dt\left(T^{test}\right)}{\alpha^{TR}\left(T^{test}\right)}$$

The stress at the testing temperature is then determined by substituting Equation (10) into Equation (9):

(11) $$\sigma =\eta \frac{d\varepsilon_{v}}{dt\left(T^{ref}\right)}=\alpha^{TR}\left(T^{test}\right)\eta \frac{d\varepsilon_{v}}{dt\left(T^{test}\right)}$$

Equation (11) indicates that temperature elevation is incorporated by multiplying the viscosity coefficient η by the shift factor $\alpha^{TR}$. As the temperature increases, the shift factor decreases, leading to faster movement in the dashpot and higher energy dissipation. Consequently, temperature elevation causes an exponential increase in dissipated energy, enhancing entropy generation and accelerating the reduction in material strength.

### 2.3. Reductions in Strength and Failure Criteria

The dissipated energies in all directions are summed up for the evaluation of entropy. The total dissipated energy is divided by the absolute temperature, yielding the generated entropy s, which is associated with strength degradation and fracture energy degradation. The strength degradations involved are shown in Table 1. Here, we evaluate the damage initiation at the $n_{0}$-th time step. $X_{T}$, $X_{C}$, $Y_{T}$, $Y_{C}$, $S_{12}$ ($=S_{13}$), and $S_{23}$ represent the strengths in the axial tensile, axial compressive, transverse tensile, transverse compressive, axial (in-plane) shear, and transverse shear directions, respectively. Subscript 0 represents their initial values. $\alpha_{AT}$, $\alpha_{AC}$, and $\alpha_{O}$ are arbitrary constants that may vary as a function of entropy s. The α ($\alpha_{AT}$, $\alpha_{AC}$, $\alpha_{A0}$) values used in this study are set three times higher than those used in [21]. A discussion of the fracture energy reduction is provided in [21]. For analyzing cracks in a cross-ply laminate, the transverse strength of the 90° ply is approximated using a Weibull distribution to achieve realistic simulations.

Furthermore, Hashin’s failure criteria are applied to evaluate damage onset and progression. Four damage initiation criteria for the fiber directional tensile, fiber directional compressive, transverse directional tensile, and transverse directional compressive modes are calculated using degraded material strengths. If any criterion is satisfied (i.e., e ≥ 1), damage onset is judged, and the damage evolution algorithm is applied. Details on updating the damage variables and stiffness tensor, accounting for material degradation after damage onset, and integrating the calculation process into Abaqus using a UMAT are provided in previous studies [20,21].

### 2.4. Material Properties and Damage Variables

Table 2 summarizes the material properties, including the parameters used for Maxwell elements n=1 to n=5. These properties comprise the elastic modulus for each spring and the viscosity for each dashpot in the Maxwell model. Table 3 lists the strength and degradation properties used in Hashin’s damage criterion to simulate damage initiation and progression.

As mentioned in Section 2.1, a time acceleration factor $\alpha_{d}$ is introduced to speed up the simulation and reduce the computational cost. Combined with the α ($\alpha_{AT}$, $\alpha_{AC}$, $\alpha_{A0}$) values, this adjustment makes the simulation 90 times faster than that reported in [21], resulting in a single simulation cycle corresponding to approximately 200 actual cycles. This approach results in discrepancies between the simulation outcomes and real values, but it ensures that all simulations are accelerated at the same rate. Therefore, these differences do not impact the qualitative analysis of the results. The non-recoverable strain parameters, as well as the entropy and damage variables, are summarized in Table 4. Finally, the thermal properties used to account for heat conduction and heat release during cyclic loading are presented in Table 5.

### 2.5. Numerical Simulation

**Figure 3:**
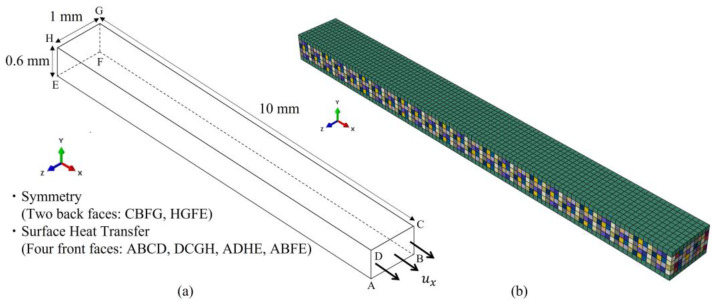
Geometric model of cross-ply CFRP laminate. (**a**) Boundary conditions, (**b**) Elements representing materials used in the simulation (green color represents the material for the 0° ply, and the other colors indicate the materials in the 90° ply).

The presented method is employed to simulate the behavior of transverse cracking in a cross-ply CFRP laminate with a stacking sequence of [0/90_4_/0] using Abaqus 2021 software. As illustrated in Figure 3a, the CFRP structure has dimensions of 10 mm × 1 mm × 0.6 mm, and the symmetrical boundary conditions are applied on surfaces CBFG and HGFE. Heat transfer is allowed to occur through four surfaces of the model, namely ABCD, DCGH, ADHE, and ABFE, which are exposed to the surrounding air, enabling interaction and thermal exchange. In the 90° ply, the transverse tensile strength is modeled using the cumulative distribution function of the Weibull distribution.

(12) $$\sigma =\sigma_{0}\left(ln\frac{1}{R-1}\right)\frac{1}{m}$$

where the shape parameter and the scale parameter (characteristic stress) $\sigma_{0}$ are set to 10 and 90 MPa, respectively. A finite element (FE) model consisting of 7777 nodes and 6000 C3D8T elements is illustrated in Figure 3b. In this model, the green color represents the material for the 0° ply, and the other colors indicate the materials in the 90° ply, with transverse tensile strength assigned based on the previously explained Weibull distribution. The element size is 0.1 mm × 0.1 mm × 0.1 mm. To maintain numerical stability, the stress boundary condition is substituted with strain boundary conditions by imposing a displacement boundary condition $u_{x}$ (corresponding to a strain of 0.5%) on surface ABCD, as illustrated in Figure 3a.

## 3. Results and Discussion

### 3.1. Numerical Results

**Figure 4:**
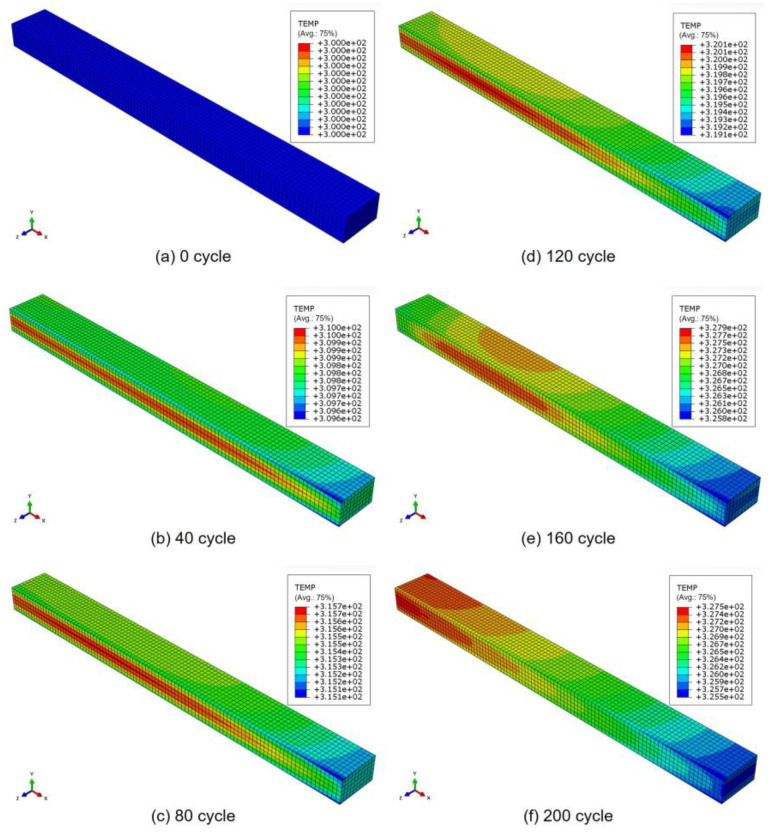
Temperature distribution in laminate with increasing fatigue cycles (at 8 Hz).

**Figure 5:**
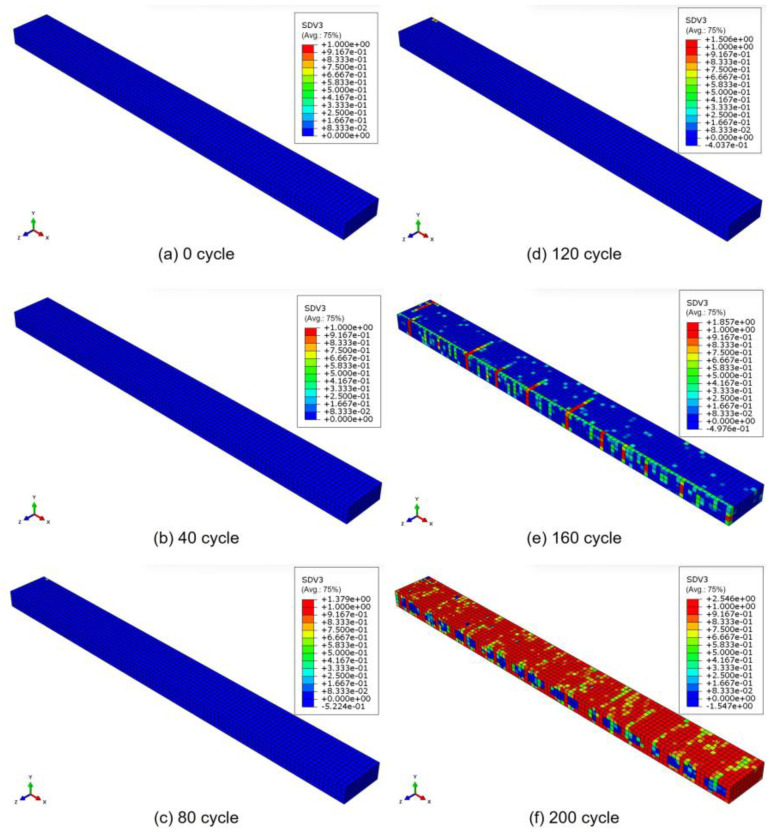
Progression of transverse cracks in 90° ply with increasing fatigue cycles (at 8 Hz).

**Figure 6:**
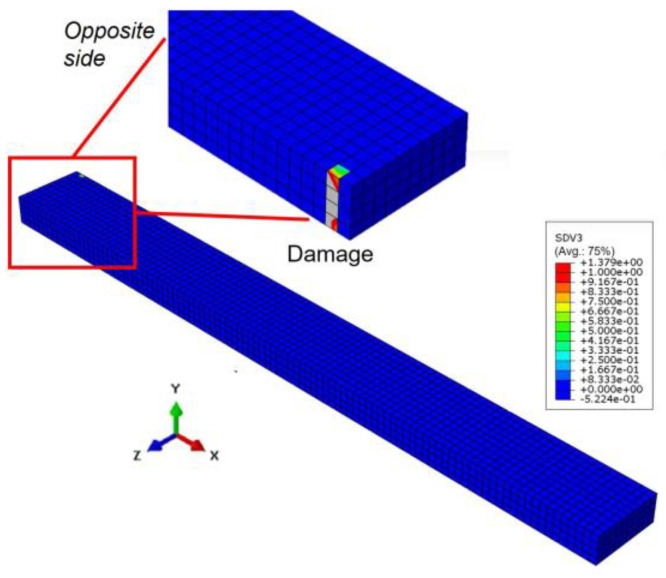
The initial damage that occurred after 100 cycles (at 8 Hz). Note that the damage evolution index is plotted, with values ranging from 0 to 1, representing the gradual transition from an undamaged to a damaged state.

Taking the results obtained at a load frequency of 8 Hz as an example, Figure 4 and Figure 5 show the temperature distribution in the laminate and the progression of transverse cracks in the 90° ply under cyclic loading, respectively. The results are shown for (a) 0 cycles, (b) 40 cycles, (c) 80 cycles, (d) 120 cycles, (e) 160 cycles, and (f) 200 cycles, indicating that both the temperature and the damage progression increase as the fatigue cycles increase. The temperature distribution reflects a realistic condition, highlighting the influence of the stacking sequence. The distributions also accurately reflect the boundary conditions (i.e., whether a symmetrical condition is applied or the surfaces allow heat transfer between the elements and the surrounding environment). Moreover, until 80 cycles, the temperature distribution follows a consistent pattern similar to the previous cycles. However, from 120 cycles onwards, the distribution changes locally, which is attributed to the effect of damage. As shown in Figure 6, damage initiates at 100 cycles, reducing the stress around the damage area. This, in turn, decreases the dissipated energy in the damaged region, ultimately leading to a localized increase in temperature compared with other areas. This phenomenon is highly realistic and challenging to capture using other analytical methods, including experiments, because conventional techniques typically measure the temperature distribution only on the material’s surface and not within its internal structure, as observed in this simulation. Experimentally, the occurrence of matrix cracks before propagating as a transverse crack, as shown in Figure 6, is a natural phenomenon and might be identified using techniques such as acoustic emission or other advanced in situ observation methods.

**Figure 7:**
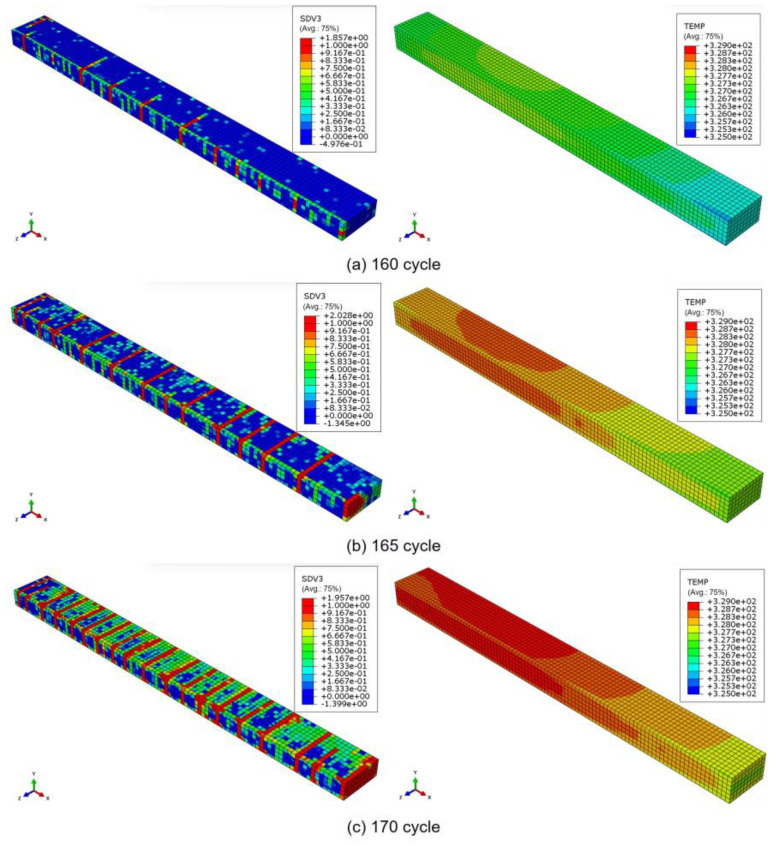
Progression of the transverse cracks (left) and temperature distribution (right) in the 90° ply at (**a**) 160, (**b**) 165, and (**c**) 170 cycles under a loading frequency of 8 Hz.

At the same loading frequency of 8 Hz, transverse cracks begin to propagate across the width (z-direction) of the model at 160 cycles, and a sudden increase in the number of transverse cracks is observed between 160 and 170 cycles. To illustrate this behavior, Figure 7 shows the progression of transverse cracks on the left and the corresponding temperature distribution on the right in the 90° ply at three stages: (a) 160 cycles, (b) 165 cycles, and (c) 170 cycles.

**Figure 8:**
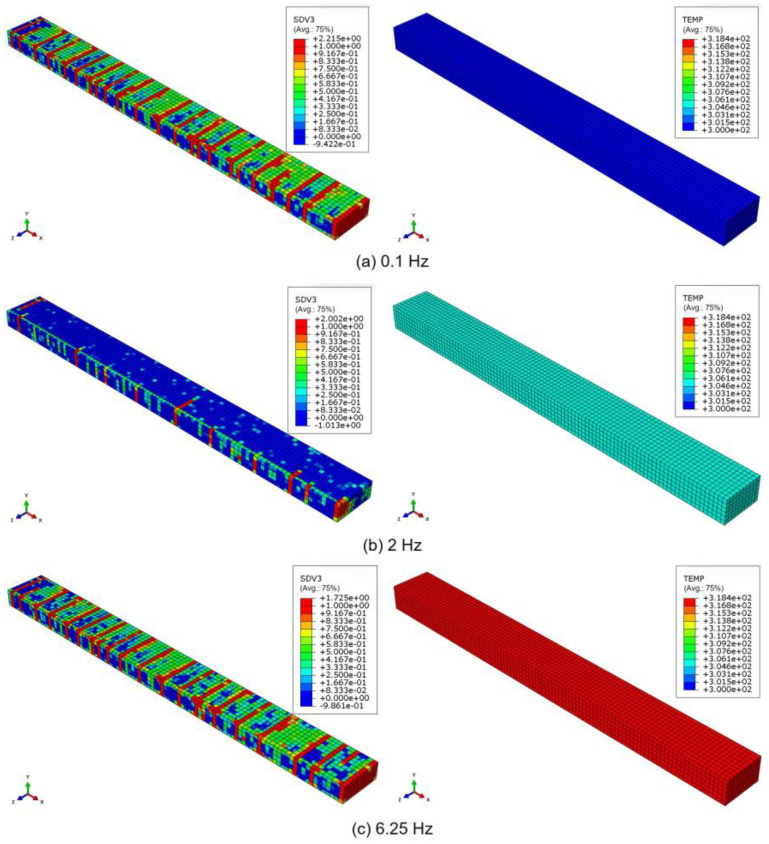
The progression of the transverse cracks (left) and temperature distribution (right) in the 90° ply after 200 cycles under a loading frequency of (**a**) 0.1 Hz, (**b**) 2 Hz, and (**c**) 6.25 Hz.

To investigate the effects of different loading frequencies, the results showing the progression of transverse cracks (left) and the corresponding temperature distribution (right) in the 90° ply after 200 cycles are presented in Figure 8 for three loading frequencies: (a) 0.1 Hz, (b) 2 Hz, and (c) 6.25 Hz. At 0.1 Hz, the temperature is the lowest, followed by that at 2 Hz, reaching its highest value at 6.25 Hz. Hence, at a low loading frequency of 0.1 Hz and a high loading frequency of 6.25 Hz, the number of transverse cracks is significantly higher, but at 2 Hz, the number of transverse cracks is comparatively much lower. At low frequencies, viscoelastic dissipation becomes more pronounced, leading to a high rate of transverse cracking. Conversely, at high frequencies, the elevated heat generation due to rapid loading contributes to a similarly high number of transverse cracks.

**Figure 9:**
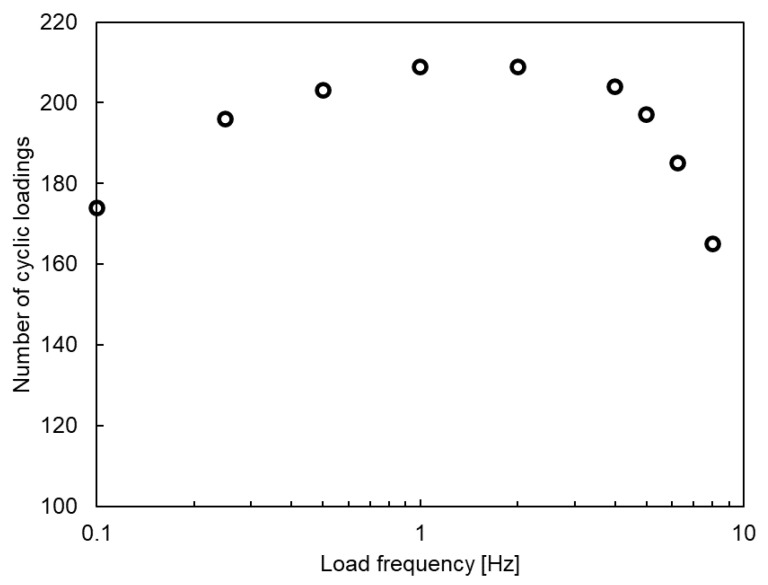
Number of cycles observed before formation of three transverse cracks versus loading frequency.

Figure 9 shows the relationship between the number of cycles observed before the formation of three transverse cracks and the loading frequency. The trend exhibits an initial increase followed by a decrease, where at low frequencies, viscoelastic dissipation is more pronounced, reducing the number of cycles required to reach a similar damage state. As the frequency increases, the number of cycles initially rises but eventually decreases owing to the effects of heat generation at higher frequencies. Specifically, as mentioned in Section 1, under high-frequency cyclic loading, the internal heat generation from dissipated energy induced during mechanical deformation, attributed to the viscoelastic and plastic behavior of the material, exceeds the thermal dissipation to the environment, leading to an increase in specimen temperature and accelerated damage progression. This is consistent with observations reported for resin materials [32], indicating that the numerical simulation successfully replicates the actual behavior. To the best of our knowledge, this is the first time such a numerical analysis has been applied to investigate damage progression in CFRP materials.

### 3.2. Comparison with Experimental Findings

**Figure 10:**
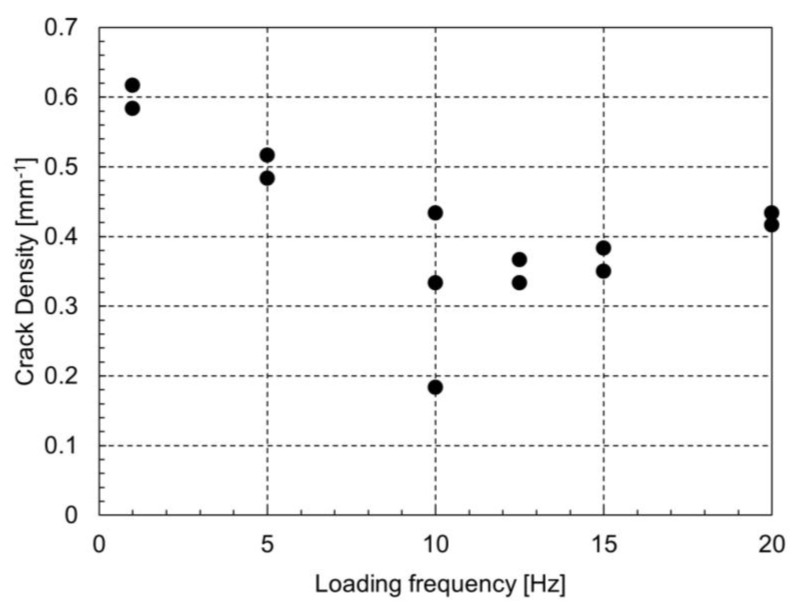
Relationship between the crack density at 10^5^ cycles and loading frequency, from experiments.

The material utilized in this study is a carbon fiber/epoxy unidirectional tape prepreg (Torayca, T700SC/2592, with a thickness of 0.14 mm per ply), and the stacking sequence of the laminate is [0/90_3_]_s_. Cracks were observed by X-ray radiography. Following the experimental setup described by Deng et al. [20], fatigue tests on the CFRP specimens were carried out at room temperature under tension–tension sinusoidal loading. The stress ratio, R, was fixed at 0.1, with the minimum stress set at 20 MPa and the maximum stress at 200 MPa (30% of the tensile strength). The experiments were conducted under the same conditions as those described in [20], with the addition of a high loading frequency (i.e., 20 Hz). Figure 10 shows the relationship between the crack density at 10^5^ cycles and loading frequency. There are some variations in the experimental results at a loading frequency of 10 Hz compared to other frequencies. Since the number of cracks in laminates under fatigue loading typically exhibits high variability due to material defects and inherent factors, these fluctuations are expected; however, further investigation using a larger number of specimens is necessary. As the loading frequency increases, the transverse crack density initially decreases and then begins to increase. This behavior qualitatively aligns with the previously mentioned analytical results. Considering that the analytical results show the number of cycles required for the appearance of three cracks, the experimental results shown in Figure 10 correspond to an inverted version of the graph shown in Figure 9. In this study, the analysis uses a damage acceleration factor, and therefore, the comparison is limited to a qualitative discussion. However, the experimental and analytical results show consistent trends.

There are several areas for further investigation highlighted by this study. First, the relationship between heat generation and dissipation warrants closer examination because this balance is dependent on the size of the specimen. The ratio of volume to surface area determines the equilibrium temperature, which must be carefully considered in future analyses. For instance, in thicker specimens, the temperature inside the specimen may be significantly higher than the surface temperature, even when surface temperature measurements (e.g., using infrared imaging) suggest otherwise. This elevated internal temperature may accelerate damage progression, potentially shifting the initiation point of transverse cracks to the interior of the specimen.

Under static loading, transverse cracks typically originate at the edges, where out-of-plane tensile stresses are severe. However, in fatigue tests, transverse cracks may originate from within the specimen because of localized internal heating. The proposed numerical model has the potential to simulate such phenomena, offering a powerful tool for capturing complex damage mechanisms. Moreover, as indicated, relying solely on surface temperature measurements to assess thermal effects can be misleading, emphasizing the need for a more comprehensive approach to characterize thermal damage behavior.

Ultimately, this study demonstrated that the progression of damage varies significantly with loading frequency. This observation underscores the importance of incorporating these findings into discussions on the fatigue damage of real structural components, particularly those with anchorage systems, as they typically experience earlier fatigue damage [51]. This work supports ongoing discussions and the development of studies on the fundamental aspects of fatigue damage in CFRP laminates, facilitating further progress in this field.

## 4. Conclusions

We conducted numerical simulations to investigate the frequency dependence of fatigue damage progression in cross-ply CFRP laminates. The initiation and propagation of transverse cracks under varying fatigue test frequencies were analyzed, revealing critical insights into the effects of loading frequency on damage behavior. The results obtained from our numerical simulation, which employs adjusted numerical values for the purpose of damage acceleration, indicate that the number of cycles required for the formation of three transverse cracks was 174 cycles at 0.1 Hz, 209 cycles at 1 Hz, and 165 cycles at 10 Hz. At high frequencies, the internal heat generation resulting from dissipated energy during mechanical deformation exceeds thermal dissipation, leading to increased specimen temperature and accelerated damage progression. Conversely, at low frequencies, viscoelastic dissipation becomes more pronounced, reducing the number of cycles required to reach a similar damage state. The rate of damage accumulation was found to initially increase with frequency before subsequently decreasing, aligning with observed fatigue phenomena. Thus, the phenomenon was successfully simulated using an enhanced degradable Hashin failure model that was originally developed by the authors in 2022. This simulation serves as the first qualitative numerical analysis of frequency-dependent fatigue damage behavior in CFRP laminates, demonstrating that the observed trend can be captured within a time-accelerated simulation space. While this study focuses on qualitative comparisons, these results pave the way for future research to refine the model through experimental validation, parameter identification, and quantitative comparisons. With further development, this approach has the potential to serve as a powerful tool for predicting and analyzing fatigue damage in composite materials, significantly contributing to the design and durability assessment of CFRP structures.

## Figures and Tables

**Table 1:** Reductions in strength, adapted from [21], Elsevier, 2022.

Strength	Equation
$\mathrm{Axial}\;\mathrm{tensile}\;X_{T}$ $\;\mathrm{and}\;\mathrm{compressive}\;\mathrm{strengths}\;X_{C\;}$	$X_{T,\;n_{0}}=\left(1-\alpha_{AT}T_{s}n_{0}\right)X_{T,\;0},\;\;\;\;X_{C,\;n_{0}}=\left(1-\alpha_{AC}C_{s}n_{0}\right)X_{C,\;0}$
Transverse tensile $Y_{T}$ and compressive strengths $Y_{C\;}$	$Y_{T,\;n_{0}}=\left(1-\alpha_{0}sn_{0}\right)Y_{T,\;0},\;\;\;\;Y_{C,\;n_{0}}=\left(1-\alpha_{O}sn_{0}\right)Y_{C,\;0}$
Shear strengths	$S_{12,\;n_{0}}=\left(1-\alpha_{O}sn_{0}\right)S_{12,\;0},\;S_{13,\;n_{0}}=\left(1-\alpha_{O}sn_{0}\right)S_{13,\;0}$
	$S_{23,\;n_{0}}=\left(1-\alpha_{O}sn_{0}\right)S_{23,\;0}$

**Table 2:** Material properties, including those of Maxwell elements.

**Elastic Modulus (MPa)**	n=1	n=2	n=3	n=4	n=5
$E_{11}^{n}$	128,000	80	80	80	80
$E_{22}^{n},\;E_{33}^{n}$	4290	267	267	267	267
$G_{12}^{n},\;G_{13}^{n}$	1810	133	133	133	133
$G_{23}^{n}$	1610	101	101	101	101
**Viscosity (MPa·s)**	n=1	n=2	n=3	n=4	n=5
$\eta_{11}^{n}$	6.00 × 10^30^	3.50 × 10^6^	3.00 × 10^6^	3.00 × 10^5^	6.00 × 10^3^
$\eta_{22}^{n},\;\eta_{33}^{n}$	6.00 × 10^30^	1.17 × 10^7^	1.00 × 10^7^	1.00 × 10^6^	2.01 × 10^4^
$\eta_{12}^{n},\;\eta_{13}^{n}$	6.00 × 10^30^	5.83 × 10^6^	5.00 × 10^6^	5.00 × 10^5^	9.99 × 10^3^
$\eta_{23}^{n}$	6.00 × 10^30^	4.45 × 10^6^	3.81 × 10^6^	3.81 × 10^5^	7.63 × 10^3^

**Table 3:** Strength and degradation properties for Hashin’s damage criterion.

Material Properties	Value
Initial axial tensile strength $X_{T,\;0}$	3930 MPa
Initial axial compressive strength $X_{C,\;0}$	2775 MPa
Initial transverse tensile strength $Y_{T,\;0}$	150 MPa
Initial transverse compressive strength $Y_{C,\;0}$	270 MPa
Initial axial shear strength $S_{12,\;0}$, $S_{13,\;0}$	117 MPa
Initial axial transverse strength $S_{23,\;0}$	117 MPa
Initial fiber directional tensile fracture energy *	12 N/mm
Initial fiber directional compressive fracture energy *	6 N/mm
Initial transverse tensile fracture energy *	0.42 N/mm
Initial transverse compressive fracture energy *	1.36 N/mm
Degradation coefficient α($\alpha_{AT}$, $\alpha_{AC}$, $\alpha_{A0}$)	3000 K·mm^3^/J
$\mathrm{Time}\;\mathrm{acceleration}\;\mathrm{factor}\;\alpha_{d}$	30

* Descriptions of these parameters are omitted in this paper to avoid an overly long explanation: please see ref. [21].

**Table 4:** Nonlinearity, non-recoverable strain, and damage variables.

Non-Recoverable Strain [32]		Damage Variables	
$\sigma_{0}$	40 MPa	ΔH	200 kJ
a	7.00 × 10^−5^	$T_{R}$	300 K
b	0.2		
c	2		
d	8		

**Table 5:** Thermal parameters.

Parameter	Value
Specific heat c	844 J/(kg∙K)
Density ρ	1550 kg/m^3^
Surface heat transfer coefficient h	20 W/(m^2^·K)
Axial thermal conductivity $\lambda_{x}$	5.47 W/(m·K)
Transverse thermal conductivity $\lambda_{y},\;\lambda_{z\;}$	0.358 W/(m·K)

## Data Availability

The original contributions presented in this study are included in the article. Further inquiries can be directed to the corresponding author.
